# Histology shows that elongated neck ribs in sauropod dinosaurs are ossified tendons

**DOI:** 10.1098/rsbl.2012.0778

**Published:** 2012-10-03

**Authors:** Nicole Klein, Andreas Christian, P. Martin Sander

**Affiliations:** 1 Steinmann Institute, Division of Palaeontology, University of Bonn, Bonn 53115, Germany; 2 Institute of Biology and Didactics, University of Flensburg, Flensburg 24943, Germany

**Keywords:** Sauropoda, histology, cervical ribs, ossified tendons, neck mechanics

## Abstract

The histology of cervical ribs of Sauropoda reveals a primary bone tissue, which largely consists of longitudinally oriented mineralized collagen fibres, essentially the same tissue as found in ossified tendons. The absence of regular periosteal bone and the dominance of longitudinal fibres contradict the ventral bracing hypothesis (VBH) postulated for sauropod necks. The VBH predicts histologically primary periosteal bone with fibres oriented perpendicular to the rib long axis, indicative of connective tissue between overlapping hyperelongated cervical ribs. The transformation of the cervical ribs into ossified tendons makes the neck more flexible and implies that tension forces acted mainly along the length of the neck. This is contrary to the VBH, which requires compressive forces along the neck. Tension forces would allow important neck muscles to shift back to the trunk region, making the neck much lighter.

## 1. Introduction

A very long neck is the hallmark of sauropod dinosaurs, the largest creatures to ever walk the Earth, and may be a key innovation for the success and gigantism of sauropods [1,2]. The long neck of sauropods could have resulted in energetically more efficient feeding [3,4]. However, the use and even the posture of sauropod necks are still very controversial [2,5]. Although some authors favoured nearly vertical neck postures and specialized high-browsing [6,7], others have argued that increased horizontal feeding range has been the primary function of the neck and that the vertical range was only limited [8,9]. The mobility of the sauropod neck is also a topic of debate (see [9–11] versus [12–15]). The posture of sauropod necks was reconstructed by several authors based on standard mechanical laws. The results are contradictory because of different interpretations of the function of mechanically relevant structures such as the cervical ribs or the assumed amount of intervertebral and zygapophyseal cartilage [10,11,13,15–19].

**Figure 1.**
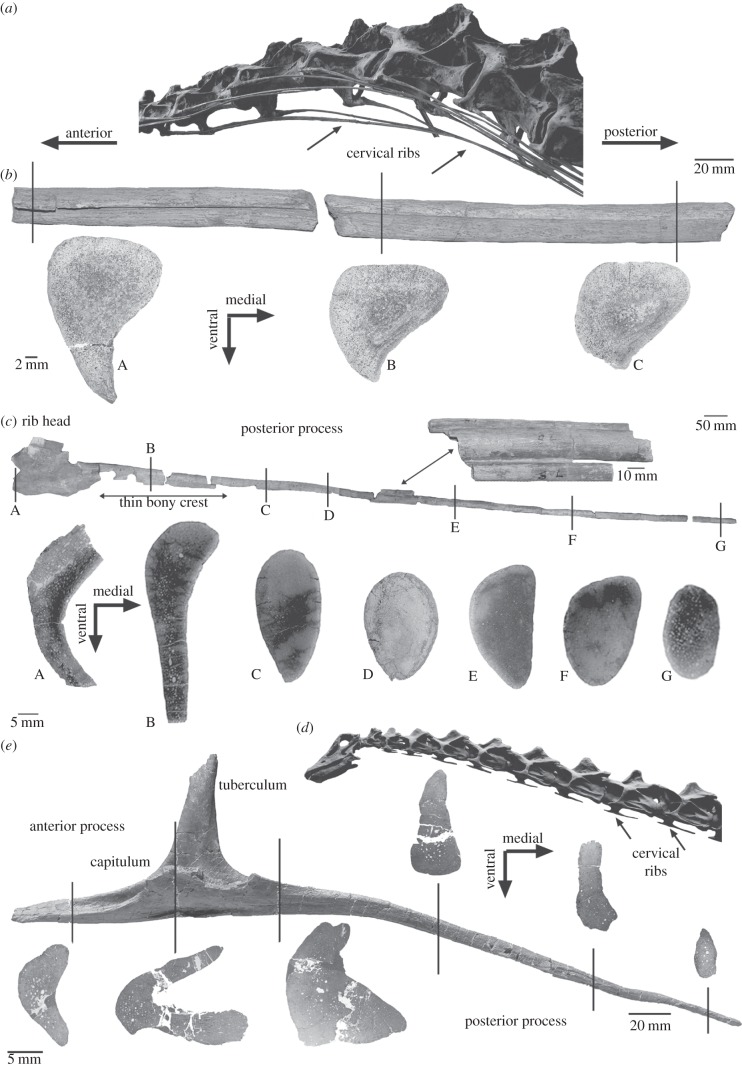
Sampled specimens, sampling locations and cross sections of sauropod cervical ribs. (*a*) Anterior neck of *Brachiosaurus brancai* (Museum für Naturkunde, Berlin) with hyperelongated and overlapping cervical ribs. (*b*) Three cross sections were taken along the proximal part of the posterior process of a left mid-neck cervical rib of *Mamenchisaurus* sp. (SIPB 597) in ventral view. Note the medially pointed ventral part of the cervical rib. (*c*) Seven cross sections were taken along the left ninth cervical rib of *B. brancai* (MB.R.2181.90), which is figured in lateral view. (*d*) Neck of *Diplodocus carnegi* (cast in the Museum für Naturkunde, Berlin) with short cervical ribs. (*e*) Six cross sections were taken along the right mid-neck cervical rib of cf. *Diplodocus* sp. (Sauriermuseum Aathal, Aathal HQ2), which is figured in ventral view. Note the morphological differences of this cervical rib when compared with the hyperelongated cervical rib of *B. brancai*.

Cervical ribs, the ribs that attach lateroventrally to the cervical vertebrae (figure 1*a*,*d*), are plesiomorphic for amniotes but are lost in some groups such as mammals. In archosauromorphs, the cervical ribs are divided into an anterior process and a posterior process (figure 1*e*). Hyperelongated cervical ribs, in which the posterior process extends backwards over several (two or three) cervical vertebrae (figure 1*a*,*c*), seem to be plesiomorphic for Sauropoda because they are already present in the sauropodomorph *Plateosaurus*. In Sauropoda, they occur, for example, in *Brachiosaurus brancai* and mamenchisaurids. In *Shunosaurus* and Diplodocoidea, the cervical ribs are shorter, and the posterior process is only somewhat longer than its corresponding vertebra [20]. Being directed posteriorly, hyperelongated cervical ribs have to overlap several other cervical ribs [21]. Shorter ribs overlap only with the anterior process of the following cervical rib, if at all.

Two competing hypotheses explain cervical rib function in sauropods: Frey & Martin [16] (see also [17]) proposed the ventral bracing hypothesis (VBH) in which the overlapping cervical ribs were bound into rods by connective tissue and supported the neck ventrally. They transferred compression forces and counteracted torque, which would have made the neck very muscular. Under the tensile member hypothesis (TMH) of Christian & Dzemski [12] (but see also [22]), the cervical ribs were used for transferring tensile forces over long distances, so that neck muscles could be shifted towards the trunk, thereby reducing the weight of the neck. The VBH implies a rather horizontal neck posture and is only reasonable for an inflexible neck, because any deviation from a maximum ventrally flexed position would have reduced the load and thereby the bracing function of the rod formed by the cervical ribs. Lateral flexion would have been largely restricted [12]. The TMH allows more flexibility of the neck and is in accordance with reconstructed dorsoventral neck mobility [12].

The aim of this paper was to test the two competing hypotheses by analysing the histology of the cervical rib of sauropods. The VBH implies loading under compression, which predicts histologically the presence of primary periosteal bone tissue. In the areas of overlapping cervical ribs, the VBH predicts connective tissue that histologically would be seen as mineralized collagen fibres oriented perpendicular to the long axis of the cervical rib. By contrast, the TMH predicts histologically the presence of longitudinally oriented mineralized collagen fibres as seen in ossified tendons of birds and some dinosaurs [23].

## 2. Material and methods

Cervical ribs of the mid-neck region of three sauropod taxa were sampled: (i) a fragmentary rib of the basal eusauropod *Mamenchisaurus* sp. (SIPB 597) from the Middle/Upper Jurassic Shaximiao Formation, Jungar Basin, China (figure 1*a*); (ii) the basal macronarian *B. brancai* (MB.R.2181.90) from the Upper Jurassic Tendaguru Formation, East Africa (figure 1*b*); and (iii) a complete mid-cervical rib of a non-*Barosaurus* diplodocine (cf. *Diplodocus* sp., SMA HQ2) from the Upper Jurassic Morrison Formation, North America (figure 1*c*). The ribs were cross-sectioned in intervals to study histological variation along their length. For comparison, ossified tendons from hadrosaurs were studied. The cross sections were processed into petrographic thin sections by standard methods [24]. Thin sections were examined with a Leica DMLP compound microscope in normal transmitted light and cross-polarized light. Collagen fibres are visible only under polarized light at high magnification and without lambda filter. Abbreviations: MB.R, Museum für Naturkunde, Berlin (Leibniz Institute for Research on Evolution and Biodiversity Berlin, Germany); SIPB, Steinmann Institute (Division of Palaeontology, Bonn, Germany); SMA, Sauriermuseum Aathal, Aathal (Canton of Zurich, Switzerland).

## 3. Results

**Figure 2.**
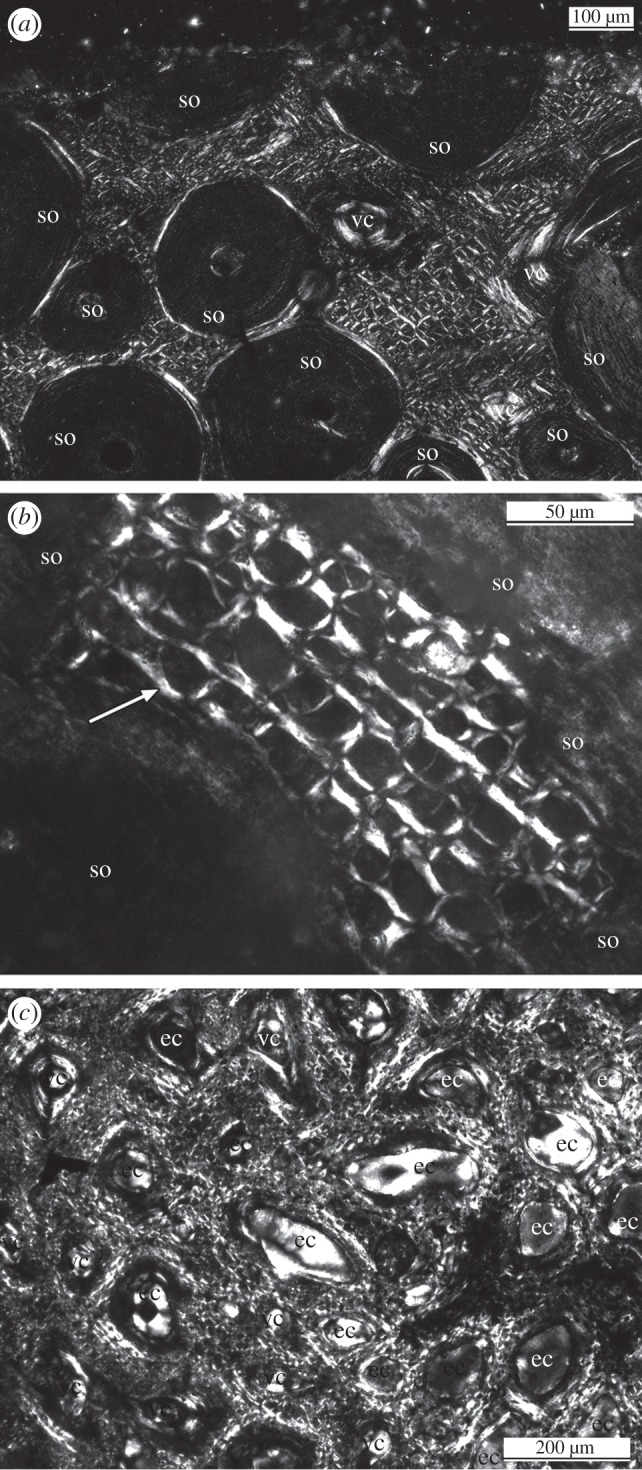
Histological details of the sampled cervical ribs of Sauropoda and an ossified tendon in polarized light. (*a*) Overview of the outer cortex of *Mamenchisaurus* sp. sample SIPB 597-C. Note the dominant longitudinal fibres between the scattered secondary osteons and the longitudinal vascular canals. (*b*) Enlargement of cf. *Diplodocus* sp. SMA HQ2-F with dense longitudinally running fibres between scattered secondary osteons. Note the diamond shape of the perpendicular cut longitudinal fibres. The fibres are surrounded by a sheath, which appears here mainly in white (arrow). (*c*) Histological detail of an ossified tendon of a hadrosaur with dense longitudinally running fibres in between erosion cavities and vascular canals. Abbreviations: ec, erosion cavity; so, secondary osteon; vc, vascular canal.

The cervical rib histology of all three taxa is dominated by secondary osteons, but primary bone can be observed in the interstices and at the outer bone surface (figure 2). The primary bone tissue shows dense, longitudinally oriented fibres organized into bundles (figure 2) instead of periosteal bone. These fibre bundles are diamond-shaped when cut exactly perpendicular and are surrounded by a distinct sheath (figure 2*b*). This tissue is of metaplastic origin, because the fibre bundles represent ossified connective tissue, i.e. tendons. Longitudinally oriented vascular canals were deposited between the fibre bundles.

The *Mamenchisaurus* sp., sample may represent an earlier ontogenetic stage because cross sections are less remodelled by secondary osteons when compared with the cf. *Diplodocus* sp., and *B. brancai* samples. *Mamenchisaurus* species shows thick layers throughout the cortex solely built of longitudinal fibres (figure 2*a*). In *B. brancai*, layers consisting solely of longitudinal fibres are also visible in the outer cortex. In cf. *Diplodocus* species, the fibres are mainly visible in the interstices between the secondary osteons and occur locally only in the outermost cortex (figure 2*b*). Generally, the intensity and amount of longitudinal fibre bundles increases posteriorly along the posterior process in all three taxa. Only in cf. *Diplodocus* sp. (SMA HQ2-A,B), the anterior process was sampled (figure 1*e*). It also shows longitudinal fibre bundles. Fibres oriented perpendicular to the rib long axis were absent in all samples.

Primary periosteal bone tissue is lacking along the posterior process, except for *Mamenchisaurus* sp., in which the ventromedial margin locally shows parallel-fibred bone tissue with simple longitudinal vascular canals and primary osteons. The rib head, which was only sampled in cf. *Diplodocus* sp., shows normal primary periosteal bone tissue with longitudinally oriented vascular canals.

The ossified hadrosaur tendons sampled for comparison consist solely of longitudinally running fibre bundles (figure 2*c*) looking exactly like those in the cervical ribs of Sauropoda (figure 2*a,b*). Ossified tendons also show longitudinal vascular canals.

We conclude that the posterior processes of the elongated and hyperelongated cervical ribs of the three sauropod taxa represent ossified tendons, which has not previously been recognized. The fact that the cervical ribs are ossified tendons allows an unequivocal interpretation of their biomechanical function.

## 4. Discussion

The predominance of longitudinal mineralized collagen fibre bundles and the lack of perpendicular fibres refute the VBH but support the TMH. Comparison with ossified tendons of hadrosaurs, birds and other dinosaurs [23] unequivocally indicates the posterior processes of the cervical ribs of *Mamenchisaurus* sp., *B. brancai* and cf. *Diplodocus* sp., are ossified tendons. Only the region around the rib heads is homologous to the plesiomorphic cervical rib proper.

The nature of sauropod cervical ribs as ossified tendons and comparative anatomical studies [22,25] suggests that the *musculus longus colli ventralis* was attached to the posterior process of the cervical rib, loading it under tension. This permitted the *musculi flexor colli* to shift posteriorly towards the trunk, making the entire neck much lighter. Ossification of the tendon probably began early in ontogeny, as is documented for other dinosaurs [23].

Our result fits the observation that hyperelongated cervical ribs are common in sauropods with extremely long necks, in which high amounts of torque would shift neck mass backward. In sauropods with shorter cervical ribs and therefore more flexible necks, the backward shift of muscle mass was limited by the need to bend the neck [13]. The predominance of tensile loading in the cervical ribs does not, however, exclude the possibility that overlapping cervical ribs contributed to bracing when the neck was kept in a low, ventrally flexed posture [21].
